# Precision of virtual surgical planning employing screw-holes locator and patient-specific implants versus conventional plate osteosynthesis for parasymphyseal mandibular fracture: a double-blinded randomized control trial

**DOI:** 10.1186/s12903-026-09303-5

**Published:** 2026-07-23

**Authors:** Ahmed Abdellatif, Hussein Hatem, Mona Elhadidy, Mohammed Omara

**Affiliations:** Department of Oral and Maxillofacial Surgery, Faculty of Dentistry, Cairo University, Cairo, Egypt

**Keywords:** Mandibular fractures, Open fracture reduction, Surgery, Computer-Assisted, Surgical Guides, Patient-Specific Implants, Virtual Surgical Planning, Randomized Controlled Trial

## Abstract

**Background:**

Conventional plate osteosynthesis is the corner stone for management of displaced parasymphyseal mandibular fractures that necessitate open reduction and internal fixation (ORIF). Yet, the conventional workflow is labor-intensive, time consuming and limited by variable reduction accuracy as it relies solely on surgeon experience. Recently, various novel computer-guided techniques have been introduced for mandibular fracture repair, hiring surgical guides of diverse designs and strategies. These techniques served as a substitute for the conventional treatment modalities, offering improved precision, speed and reproducibility. Therefore, this randomized controlled trial (RCT) compares the overall precision of this distinct complete computer-guided virtual surgical planning (VSP) protocol as a whole, employing screw-holes locator and custom-made plates versus the conventional workflow for parasymphyseal mandibular fracture repair.

**Methods:**

Twenty-six cases presented with uncomminuted parasymphyseal mandibular fracture were enrolled and randomized into two groups. The interventional group underwent ORIF utilizing a screw-hole locator and custom-made plates, while the control group utilized ready-made mini-plates for ORIF. In both groups, virtual surgical planning of the fractured mandible was accomplished, creating a virtual reduced model by Mimics software aided by preoperative computer tomography (CT) scan. Then, three-dimensional model derived from the postoperative CT scan was superimposed onto the VSP reduced model. Linear deviations between predefined dental and bony landmarks to established anatomical planes were measured and underwent statistical analysis.

**Results:**

The actual postoperative mandibular model in the computer-guided group demonstrated negligible deviation from the VSP model. While the conventional group revealed considerably greater deviation. The average deviation of the bony landmarks was 0.044 ± 0.14 mm for the intervention group versus 0.69 ± 0.5 mm for the control group (*p* < 0.001). The average deviation of the dental landmarks was 0.04 ± 0.13 mm for the intervention group versus 0.47 ± 0.33 mm for the control group (*p* < 0.001). The average surgical time of the intervention group was 1.46 ± 0.18 h versus 1.80 ± 0.34 h for the control group (*p* = 0.004), with the intergroup difference being statistically significant.

**Conclusions:**

The integration of screw-holes locator and patient-specific implants (PSI) have improved anatomical reduction precision, facilitated reestablishment of the patient’s native occlusion and ensured more standardized reproducible outcomes, especially beneficial for less experienced surgeons.

**Trial registration:**

The study is a randomized control trial, registered at ClinicalTrials.gov Protocol Registration and Results System Receipt, NCT05647460, Data: 2022–12-05.

## Introduction

The parasymphysis is an ill-defined region between the symphysis and the mental foramen, lies within the interforaminal zone, and often arbitrarily defined as the region between the midline and a line corresponding to the longitudinal axis of the mandibular canine tooth [1]. Parasymphyseal fractures are usually vertical or oblique fractures that occur between the canine and lateral incisor, or between the lateral and central incisors [2]. According to Champy's biomechanical principles, parasymphyseal fractures are subjected to complex counteracting forces including tension forces along the superior alveolar border and compression forces along the inferior border. Therefore, open reduction and internal fixation (ORIF) is the well-established method for management of parasymphyseal mandibular fracture, utilizing two malleable miniplates placed along the Champy’s ideal lines of osteosynthesis. One superior miniplate placed along the alveolar border (tension band) and one inferior miniplate placed along the inferior border of the mandible (compression band). This two-plate configuration neutralizes both tensile and compressive forces, providing optimal stability for fracture healing [3].

The surgical management of parasymphyseal fracture is complicated by several anatomical and biomechanical factors. The primary challenge is counteracting the bending forces exerted by the suprahyoid musculature, which commonly tend to displace the anterior segment inferiorly, posteriorly and medially [4]. Additional technical concerns include iatrogenic injury to the roots of the canine and incisor teeth during hardware fixation, and the inherent risk of infection associated with fractures communicating with the oral flora. Inadequate stabilization can lead to significant complications, including malunion, non-union, and persistent postoperative malocclusion [5]. Malunion occurs in 0–4.2% of fractures due to inadequate reduction, inadequate immobilization, and improper application of rigid internal fixation [6].

However, the primary drawback of the conventional workflow is the inadequate exposure of the lingual plate specifically in compound sagittal oriented fractures [4]. This constrained surgical field can hinder accurate three-dimensional (3D) anatomical reduction leading to lingual cortex flaring, which can lead to serious postoperative complications including anatomical abnormalities, cosmetic deformities, and persistent functional impairments. These complications may require further orthodontic rehabilitation or revision corrective surgeries [7, 8].

Ready-made miniplates are the workhorse of mandibular trauma, offering speed, cost effectiveness, and proven reliability for the vast majority of routine fractures [9]. Accordingly, prefabricated mini-plates systems are manufactured from titanium grade 23 (Ti-6Al-4V ELI). Titanium grade 23 (Ti-6Al-4V ELI) has very high strength (ultimate tensile strength = 900–1000 MPa), moderate ductility, and fatigue resistance [10].

Recently, virtual surgical planning (VSP) has transformed the preoperative management in the maxillofacial field. Surgeons can now utilize 3D computed tomography (CT) data to perform precise digital osteotomies and anatomical reconstructions prior to entering the operating theatre [11]. In mandibular traumatology, VSP protocols aided by 3D printing and Computer aided design/computer aided manufacturing (CAD/CAM) technologies were used in various studies to introduce patient-specific surgical guides. These guides have transferred the VSP to the operative field, facilitating an accurate mandibular fracture reduction, and the subsequent application of the traditional fixation hardware [12–16].

Therefore, the present study aimed to evaluate the overall precision of a distinct complete computer-guided virtual surgical planning protocol, employing a screw-hole locator and patient-specific plates osteosynthesis compared to conventional plate osteosynthesis for parasymphyseal mandibular fracture repair. The goal was to evaluate the integrated digital-analogue workflow as a whole rather than to isolate the effect of any single component.

## Materials and methods

### Trial design and setting

**Fig. 1:**
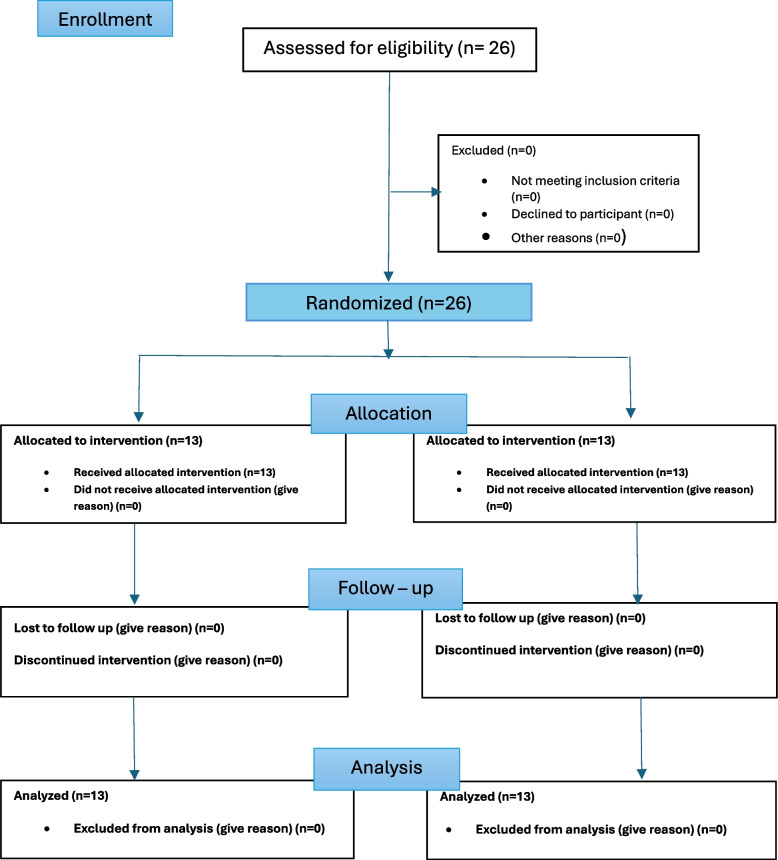
CONSORT flow diagram. Twenty-six patients with isolated parasymphyseal mandibular fractures were randomized equally into two groups: study group (computer-guided, *n* = 13) and control group (conventional, *n* = 13). All participants received the allocated intervention, completed the trial, and were included in the analysis. No losses or exclusions occurred

This prospective, parallel group, double-blinded randomized controlled trial (RCT) registered 26 consecutive patients from the Department of Oral and Maxillofacial Surgery’s clinic within the Faculty of Dentistry, Cairo University. The methodology and reporting adhered to the Consolidated Standards of Reporting Trials (CONSORT) guidelines (Fig. 1). Ethical approval was acquired from the institutional review board (IRB) at Cairo University (Approval No: 3922, date: 27 September 2022). This clinical investigation was prospectively registered in the ClinicalTrials.gov protocol registry under the identifier: NCT05647460, Data: December 9, 2022.

### Participants (eligibility criteria)

#### Inclusion criteria

The chief inclusion criteria in the present RCT were participants aged 18 to 50, of any gender, presenting with an isolated uncomminuted parasymphyseal mandibular fracture requiring ORIF. Also, dentate and partial edentulous patients with intact lower centrals incisors and sufficient posterior occluding pairs.

#### Exclusion criteria

Exclusion criteria included cases with maxillary fractures, mandibular fractures resulting from an underling Pathological lesion, an attenuated mandible, fully edentulous patients, patients undergoing chemotherapy or radiotherapy, and patients with missing lower central incisors or partial edentulous cases with insufficient posterior occluding pairs required for accurate radiographic assessment of the occlusion.

### Interventions

#### Preoperative patient’s assessment

All participants underwent a comprehensive clinical examination including a review of complete dental and medical history. To ensure eligibility, clinical measurements were obtained and assessed against the predefined inclusion criteria before proceeding with subsequent management procedures.

All participants underwent a preoperative 1:1 magnification orthopantogram (OPG) as an initial screening of the region of interest for any pre-existing pathological lesions or signs of segmental bone loss. Subsequently, a preoperative CT scan was obtained for VSP.

#### Preoperative preparation

##### In the study group

**Fig. 2:**
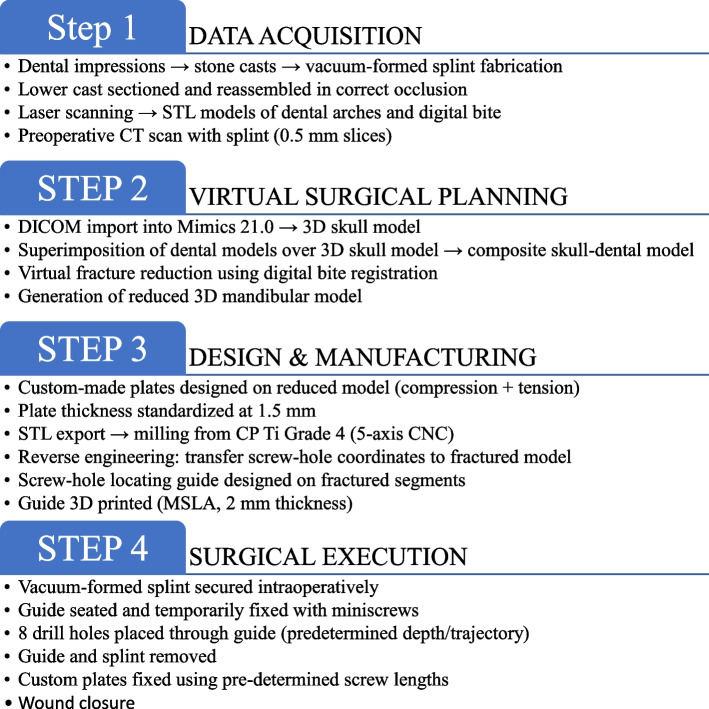
Flowchart of the study group methodology

**Fig. 3:**
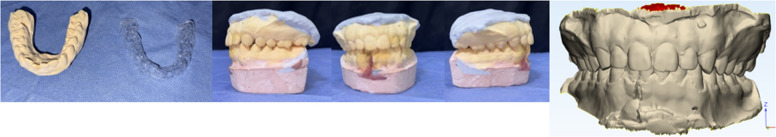
Case (1): preoperative photograph showing the vacuum-formed splint, the dental stone casts, their corrected digital bite

**Fig. 4:**
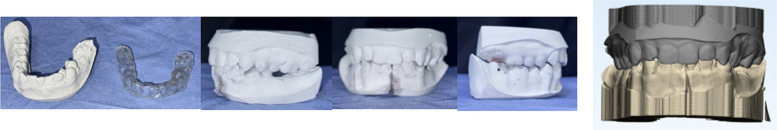
Case (2): preoperative photograph showing the vacuum-formed splint, the dental stone casts, their corrected digital bite

The digital workflow for the study group is summarized in (Fig. 2). Briefly, dental impressions and subsequent dental casts were acquired, and a vacuum-formed splint was fabricated on the lower dental cast. The lower dental cast was then sectioned along the fracture line and reassembled in correct dental alignment to achieve centric occlusion in relation to the upper cast. Both dental casts and their associated bite registration were laser scanned using a 3D scanner (Medit i600; Medit Corporation, Seoul, South Korea) to convert the stone dental casts into STL virtual models and their corresponding digital bite (Figs. 3 and 4).

**Fig. 5:**
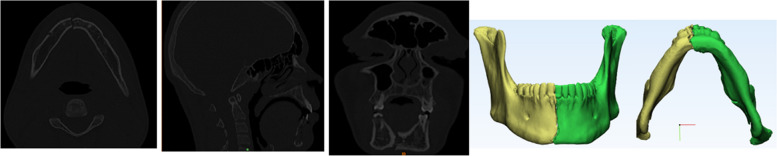
Case (1): Snapshot of multiplanar Mimics (Materialise NV) screen showing the axial, coronal, sagittal and 3D of fractured mandible

**Fig. 6:**
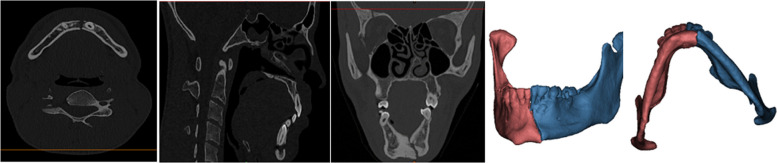
Case (2): Snapshot of multiplanar Mimics (Materialise NV) screen showing the axial, coronal, sagittal and 3D of fractured mandible

**Fig. 7:**
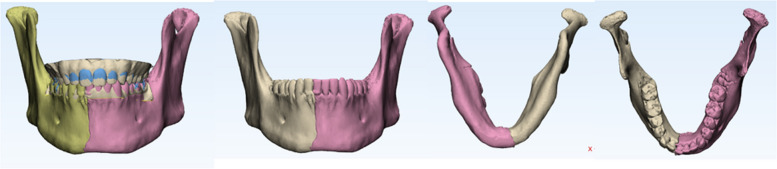
Case (1): Snapshot of virtual reduction of the mandibular model using the corrected digital bite

**Fig. 8:**
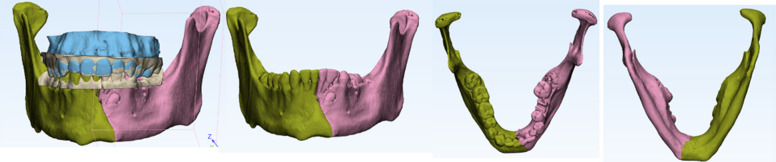
Case (2): Snapshot of virtual reduction of the mandibular model using the corrected digital bite

A preoperative CT scan was then acquired while the patient wore the previously fabricated vacuum-formed splint, using a TOSHIBA Alexion multislice unit (Toshiba Medical Systems Corporation, USA; model year 2011) (0.5 mm slice thickness, 0.3 mm reconstruction interval). The Digital Imaging and Communications in Medicine (DICOM) format were imported to Mimics 21.0 (Materialise N.V., Leuven, Belgium) to create a 3D skull model through segmentation with appropriate bony threshold (Figs. 5 and 6). This model was subsequently superimposed onto the dental models to create composite skull-dental model. VSP was done on this composite skull-dental model, which was then translated and superimposed over the predetermined digital bite registration to generate a reduced 3D virtual mandibular model (Figs. 7 and 8). This model was later used for comparison to the actual skull model derived from the postoperative CT scan for the radiographic statistical analysis.

**Fig. 9:**
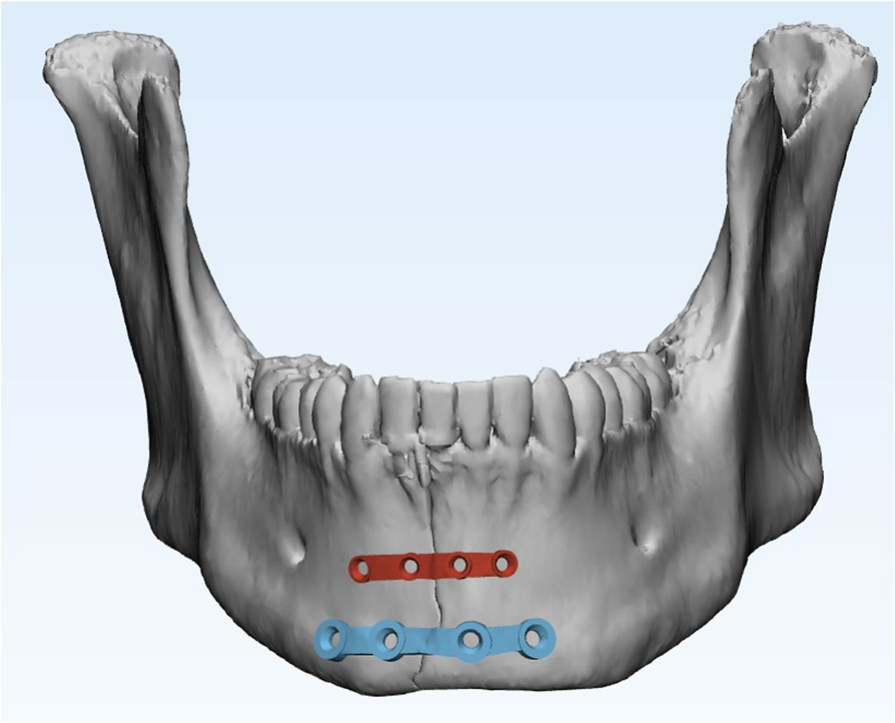
Case (1): Snapshot of multiplanar Mimics (Materialise NV) screen showing the virtually reduced mandible model with the designed custom-made plates

**Fig. 10:**
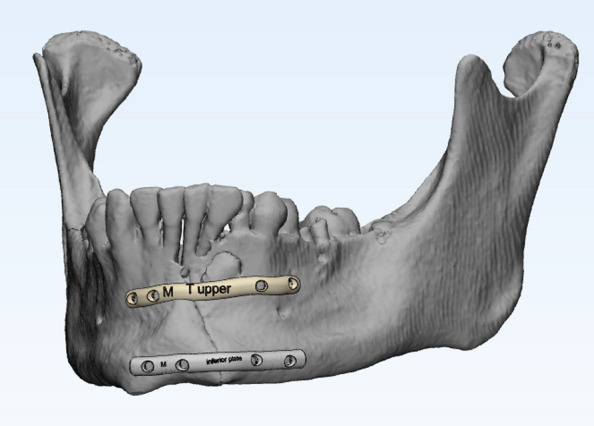
Case (2): Snapshot of multiplanar Mimics (Materialise NV) screen showing the virtually reduced mandible model with the designed custom-made plates

Two custom-made plates were then designed on the virtually Corrected mandibular model along the compression and tension bands, and exported via Stereolithographic (STL) files for milling (Arab Engineers for Designs and Medical Instrumentation, Assiut, Egypt) from commercially pure titanium (CP Ti) grade 4 blanks using a Mill star 3D 5-axis Computer Numerical Control System (CNC) milling machine (Taiwan) (Figs. 9 and 10). Plate thickness was standardized at 1.5 mm for all cases to ensure uniformity in fixation hardware. Variations in plate design (contour, length, screw hole spacing), reflect patient-specific adaptations to individual fracture patterns and anatomical morphology rather than differences in design methodology or designer.

**Fig. 11:**
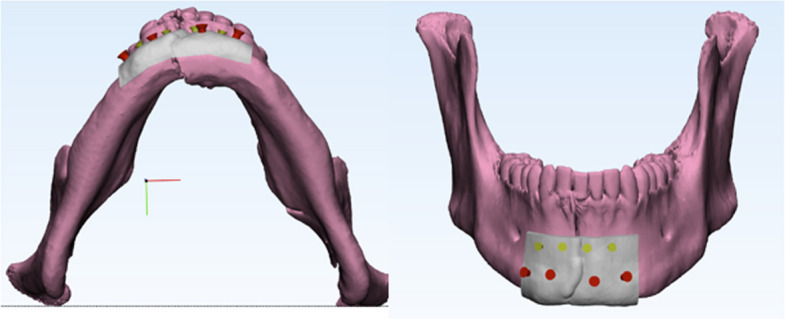
Case (1): Snapshot of the multiplanar Mimics (Materialise NV) screen showing the designed screw-holes locator adapted on the fractured mandible

**Fig. 12:**
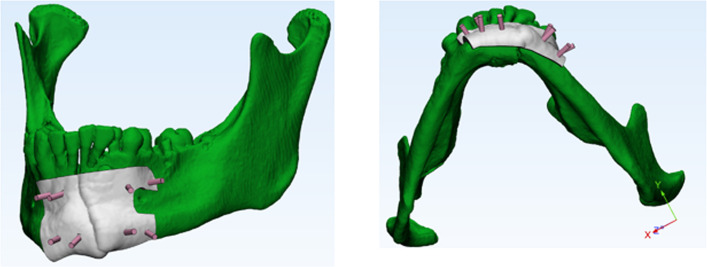
Case (2): Snapshot of the multiplanar Mimics (Materialise NV) screen showing the designed screw-holes locator adapted on the fractured mandible

The precise screw-hole imprints from the designed plates over the virtually reduced model were then transported to the fractured segments in the trauma position to design the screw-holes locator using digital reverse engineering steps, and exported via STL for 3D printing (Figs. 11 and 12). This guide was additively manufactured from resin using an Anycubic Photon M3 4 K printer, which employs masked stereolithography technology. Guide thickness was standardized at 2 mm for all cases.

The senior author (an experienced maxillofacial surgeon and member of the surgical team) designed all the custom-made plates and the screw-holes locators, aided by a specialized biomedical engineer at Arab Engineers for Designs and Medical Instrumentation. These patent’s specific implants (PSI) and guides cost approximately 3600–4000 Egyptian pound.

##### In the control group

**Fig. 13:**
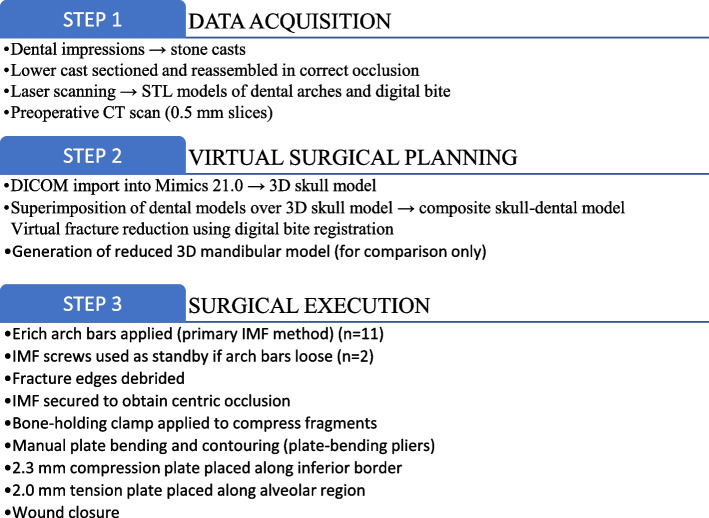
Flowchart of the control group methodology

The digital workflow for the control group is summarized in (Fig. 13). Briefly, each patient underwent a preoperative CT scan using TOSHIBA Alexion multislice unit. The DICOM format were imported to Mimics 21.0 to create 3D skull model through segmentation with appropriate bony threshold.

Dental impression and subsequent dental casts were acquired. The lower dental cast was sectioned along the fracture line and reassembled in correct dental alignment to achieve centric occlusion in relation to the upper cast. Both dental casts and their associated bite registration were laser scanned using a 3D scanner (Medit i600) to generate STL models of the dental arches and their corresponding digital bite. These were subsequently superimposed onto the skull model to create composite skull-dental model.

VSP was done on this composite skull-dental, which was then translated and superimposed over the predetermined digital bite to generate a reduced 3D virtual mandibular model. This model was later used for comparison to the actual skull model derived from the postoperative CT scan for the radiographic statistical analysis.

### Timeline from hospital presentation to surgery and preoperative preparations summarized in (Table 1)

**Table 1:** Timeline from hospital presentation to surgery and preoperative preparations

Parameter	Study Group (Computer-Guided)	Control Group (Conventional)
Timeline from presentation to surgery	3–5 days	3–5 days
Preoperative laboratory tests	Complete blood count, coagulation profile, blood grouping, random blood sugar	Complete blood count, coagulation profile, blood grouping, random blood sugar
Anesthesia evaluation	Pre-anesthesia consultation, airway assessment, ASA classification	Pre-anesthesia consultation, airway assessment, ASA classification
Dental impressions and cast fabrication	Yes, with vacuum-formed splint fabrication in trauma position	Yes, without splint
Interim fracture stabilization	Vacuum-formed splint placement	Erich arch bars (primary) or IMF screws (standby if arch bars loose)
Virtual surgical planning	Yes, for designing custom-made plates and screw-holes locator, and for comparison	Yes, for comparison only (no guide/plate design)
Digital workflow time	24 h (CT import to STL export of guides and plates)	Not applicable
Device fabrication	Custom plates (milled from CP Ti Grade 4) + surgical guides (3D printed)	Not applicable
Device sterilization and delivery	1–2 additional days	Not applicable

#### Surgical procedure

##### In both groups

All surgical steps were executed under general anesthesia via nasotracheal intubation. To achieve local hemostasis and postoperative analgesia, Articaine hydrochloride 4% with epinephrine 1:100,000 (Septodont, France) was administered along the labial sulcus incision line. The fracture was exposed by an intraoral vestibular incision, saving a healthy and adequate soft tissue cuff to help with closure and minimize the risk of dehiscence. The mental nerve was identified and carefully dissected to avoid iatrogenic neurosensory injury and permit wide flap retraction safely. Consequently, minimal retraction was required for visualization and subsequent surgical procedures.

##### Study group

**Fig. 14:**
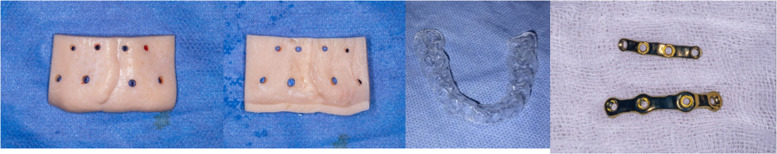
Case (1): Intraoperative clinical photograph showing the execution tools for computer-guided group: custom-made plates, screw-holes locator and the vacuum-formed splint

**Fig. 15:**
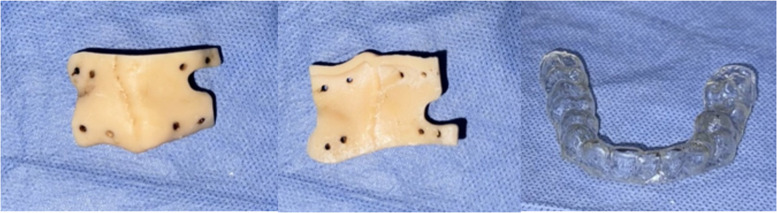
Case (2): Intraoperative clinical photograph showing the execution tools for computer-guided group: custom-made plates, screw-holes locator and the vacuum-formed splint

The prepared implementation devices applied were the custom-made plates, screw-holes locator, vacuum-formed splint, and screws kit (Figs. 14 and 15).

Prior to surgical procedures, the vacuum-formed splint was secured intraoperatively over the dentition to secure the fractured segment’s anatomical and spatial relationships in the trauma position identical to the position derived from the preoperative CT acquisition, upon which the screw-holes locator was fabricated during the VSP workflow.

**Fig. 16:**
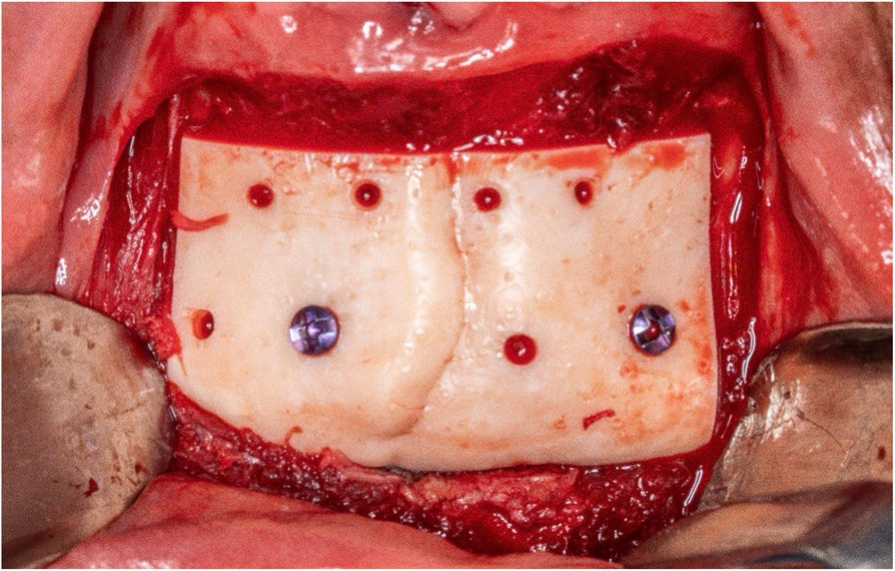
Case (1): Intraoperative clinical photograph showing the screw-holes locator adapted on the mandible and the vacuum-formed splint

**Fig. 17:**
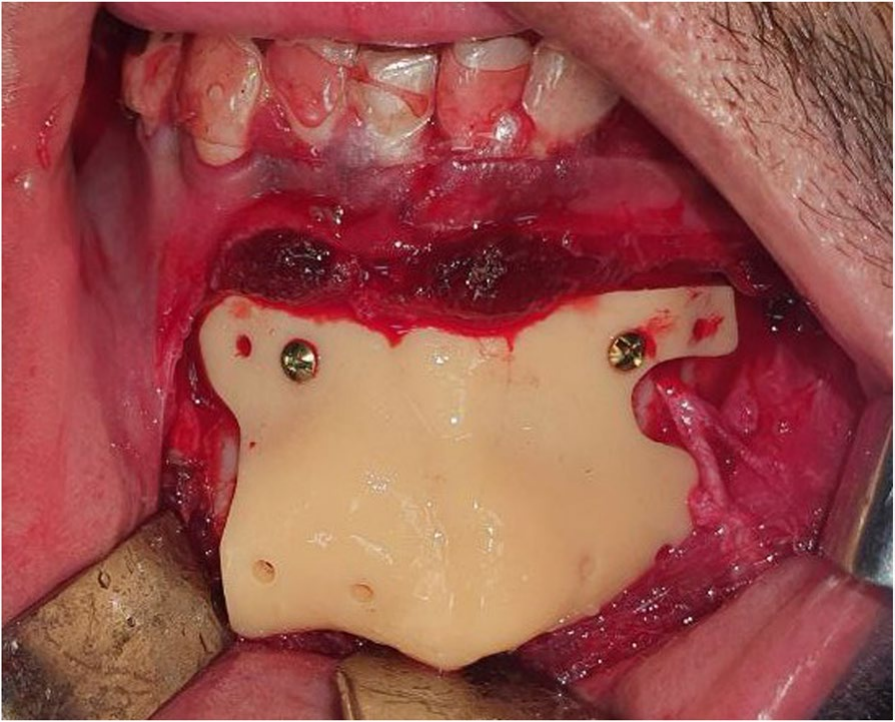
Case (2): Intraoperative clinical photograph showing the screw-holes locator adapted on the mandible and the vacuum-formed splint

The screw-holes locator was then installed and adapted onto the fractured mandible using finger pressure, to achieve passive adaptation (Figs. 16 and 17). Temporary fixation screws were then placed through designated pilot holes in the guide into stable bone segments proximal and distal to the fracture. This rigidly fixed the guide to the mandible before any drill holes were drilled. Visual and tactile confirmation of complete guide-to-bone contact was performed along the entire guide perimeter before proceeding. A meticulous periosteal elevation was performed to ensure the bone surface was fully exposed and free of soft tissue interposition at all guide contact points. The guide design intentionally avoided areas with significant overlying mentalis muscle attachments to minimize lift-off forces.

**Fig. 18:**
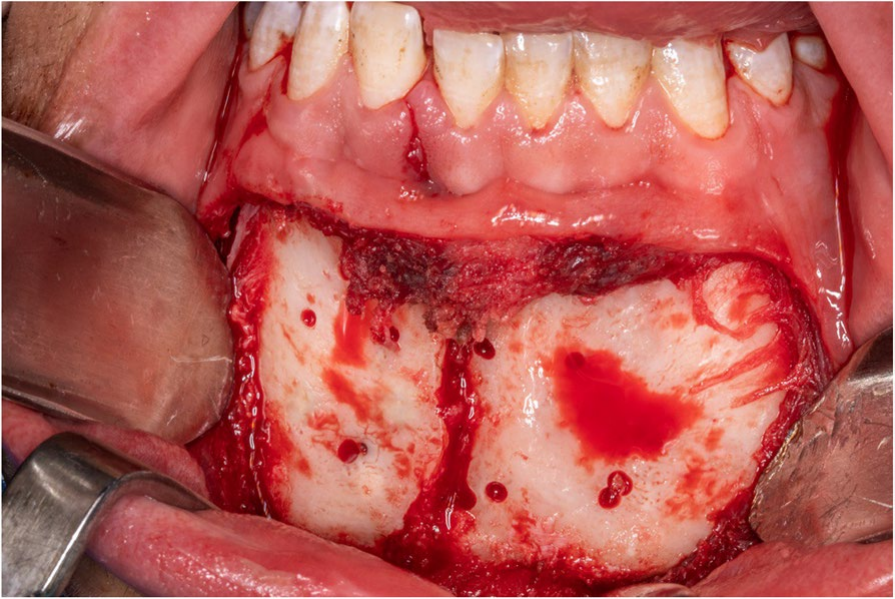
Case (1): Intraoperative clinical photograph showing 8 drill holes for the custom-made plates osteosynthesis

**Fig. 19:**
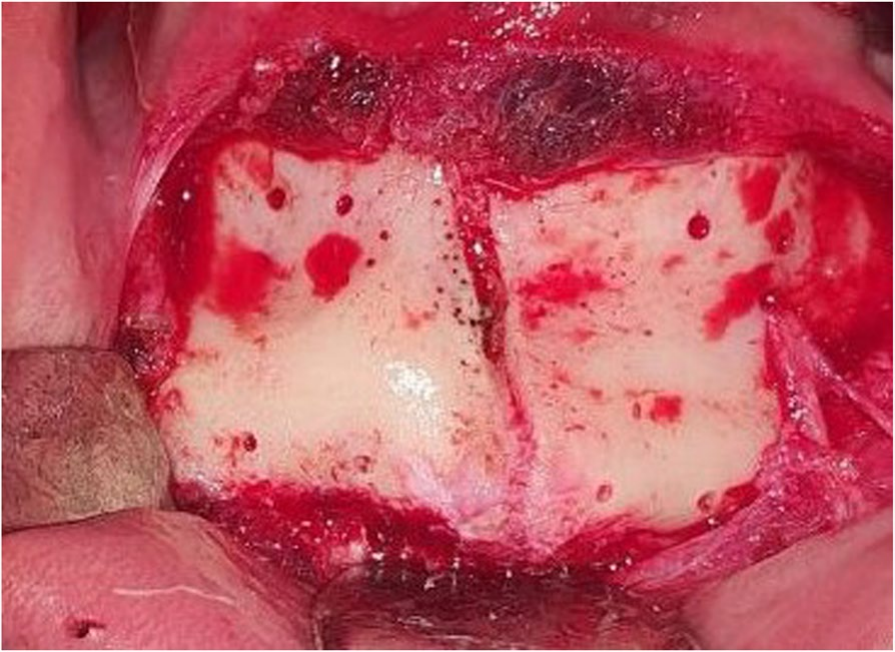
Case (2): Intraoperative clinical photograph showing 8 drill holes for the custom-made plates osteosynthesis

**Fig. 20:**
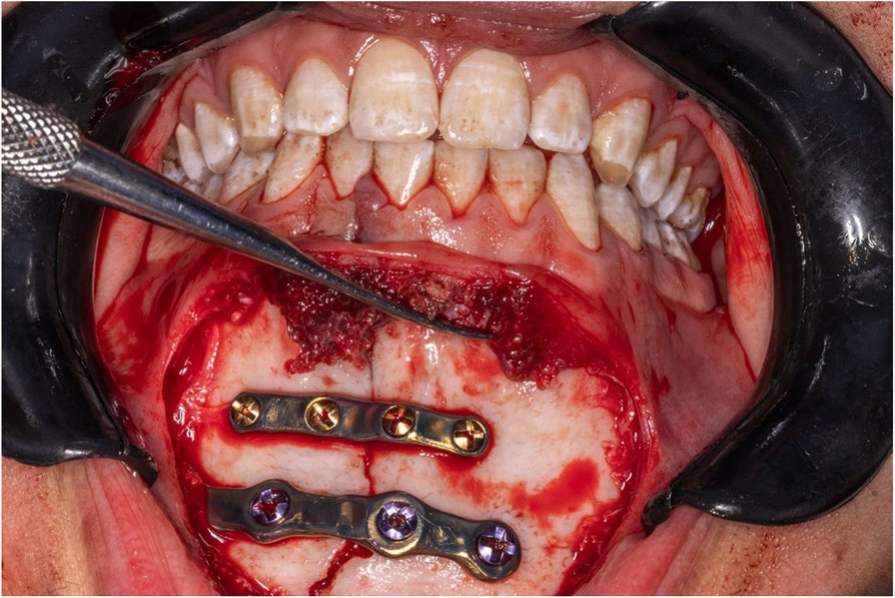
Case (1): Intraoperative clinical photograph showing custom made plates osteosynthesis

**Fig. 21:**
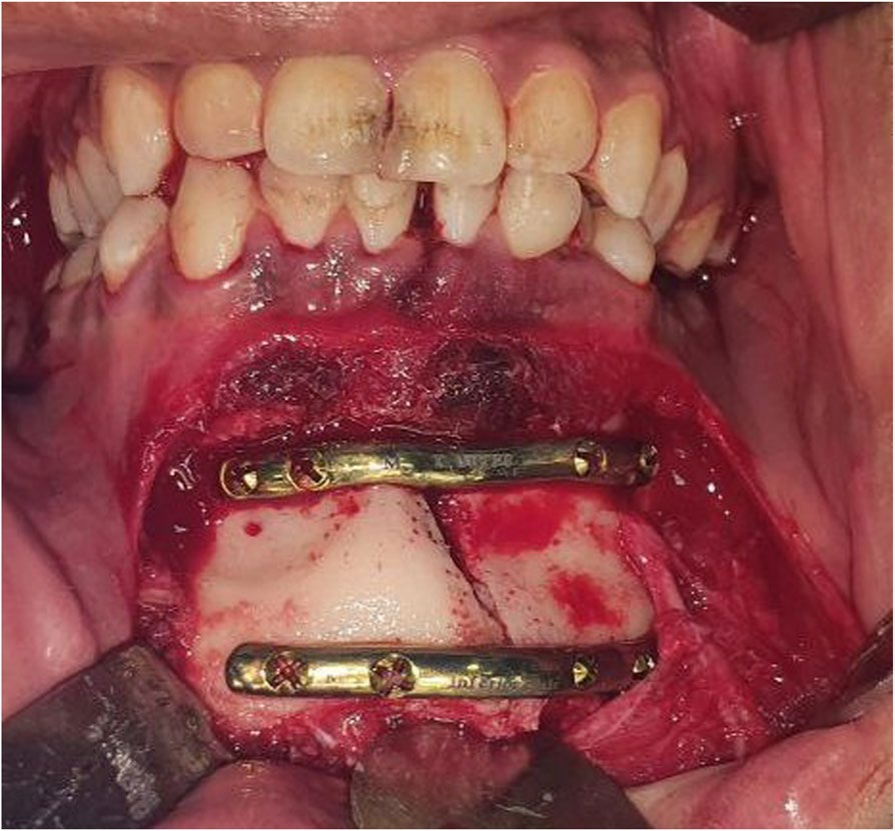
Case (2): Intraoperative clinical photograph showing custom made plates osteosynthesis

Through this guide, eight drill holes of predetermined trajectories and depth were drilled (Figs. 18 and 19). The guide and splint were then detached. The fracture edges were debrided to remove any intervening fibrous tissue or callus. Subsequently, the fracture segments were anatomically realigned under the guidance of the computer-guided custom-made plates (Figs. 20 and 21). These plates were fixated precisely using screws of optimal, predetermined lengths that fits into their corresponding drill holes. Finally, wound closure was achieved with a continuous running suture of 4–0 polyglycolic acid (Vicryle; Ethicon, Inc.).

##### Control group

Prior to surgical procedures, Erich arch bars were applied to both dental arches. Inter maxillary fixation (IMF) screws was used as an alternative in two cases where Erich arch bars lacked sufficient stability and firmness across the fractured segments. To remove any intervening fibrous tissue or callus, the fracture edges were debrided. Then to obtain an intermaxillary centric relationship, IMF was firmly secured. The fractured segments were compressed together across the fracture interface by a bone-holding clamp. After anatomical reduction was obtained, plate-bending pliers were applied to each plate for optimal contouring and adaptation of the miniplates to the patient-specific anatomical curvature of the mandible.

The fractured segments were fixated by two ready-made miniplates along the Champy’s ideal lines of osteosynthesis. Compression 2.3 miniplate (Arab Engineers for Designs and Medical Instrumentation, Assiut, Egypt; titanium grade 23, Ti-6Al-4V ELI) was initially applied, followed by tension 2.0 miniplate. Finally, wound closure was achieved with a continuous running suture of 4–0 polyglycolic acid (Vicryle; Ethicon, Inc.).

### Outcomes

#### Primary outcome (precision of reduction) (Fig. 22)

**Fig. 22:**
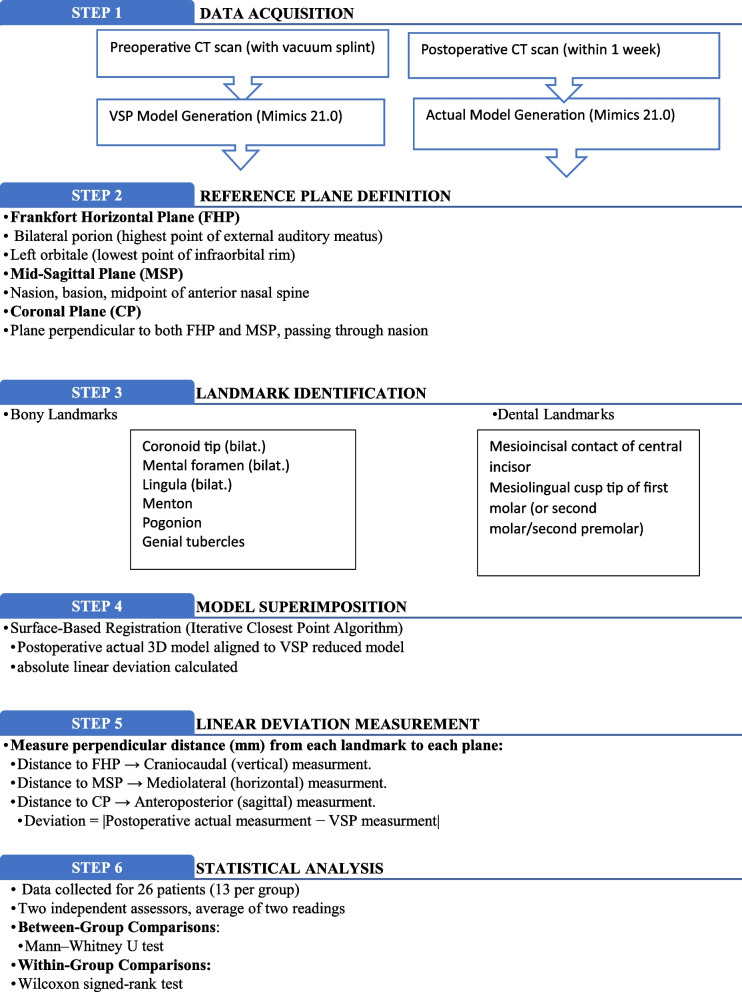
Flowchart illustrating the primary (precision of anatomical reduction) and secondary (occlusion) outcomes radiographic assessment methodology

**Fig. 23:**
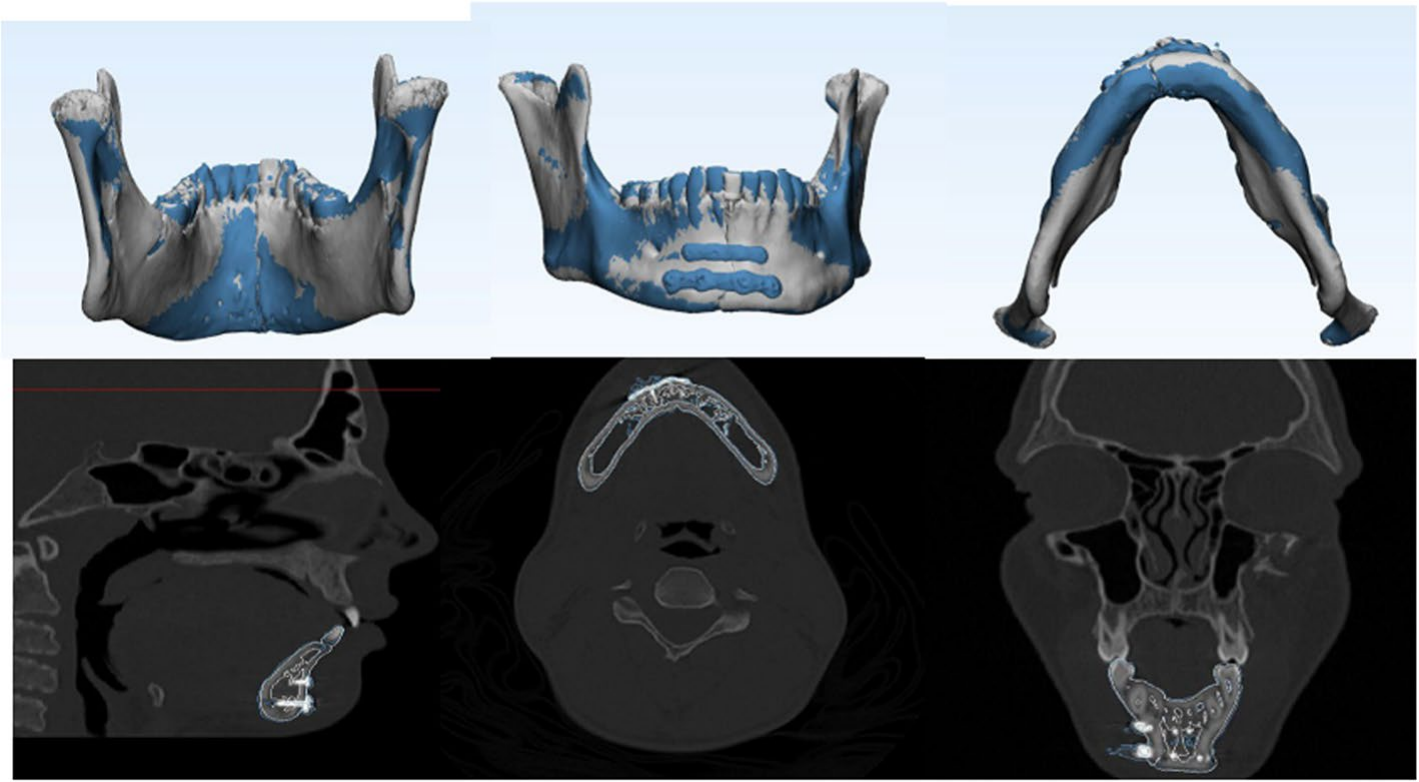
Case (1): Snapshot of multiplanar Mimics (Materialise NV) screen showing the post operative radiographic assessment (2D and 3D congruence) through super imposition of the actual postoperative CT model over the virtual surgical planning model in the study group

**Fig. 24:**
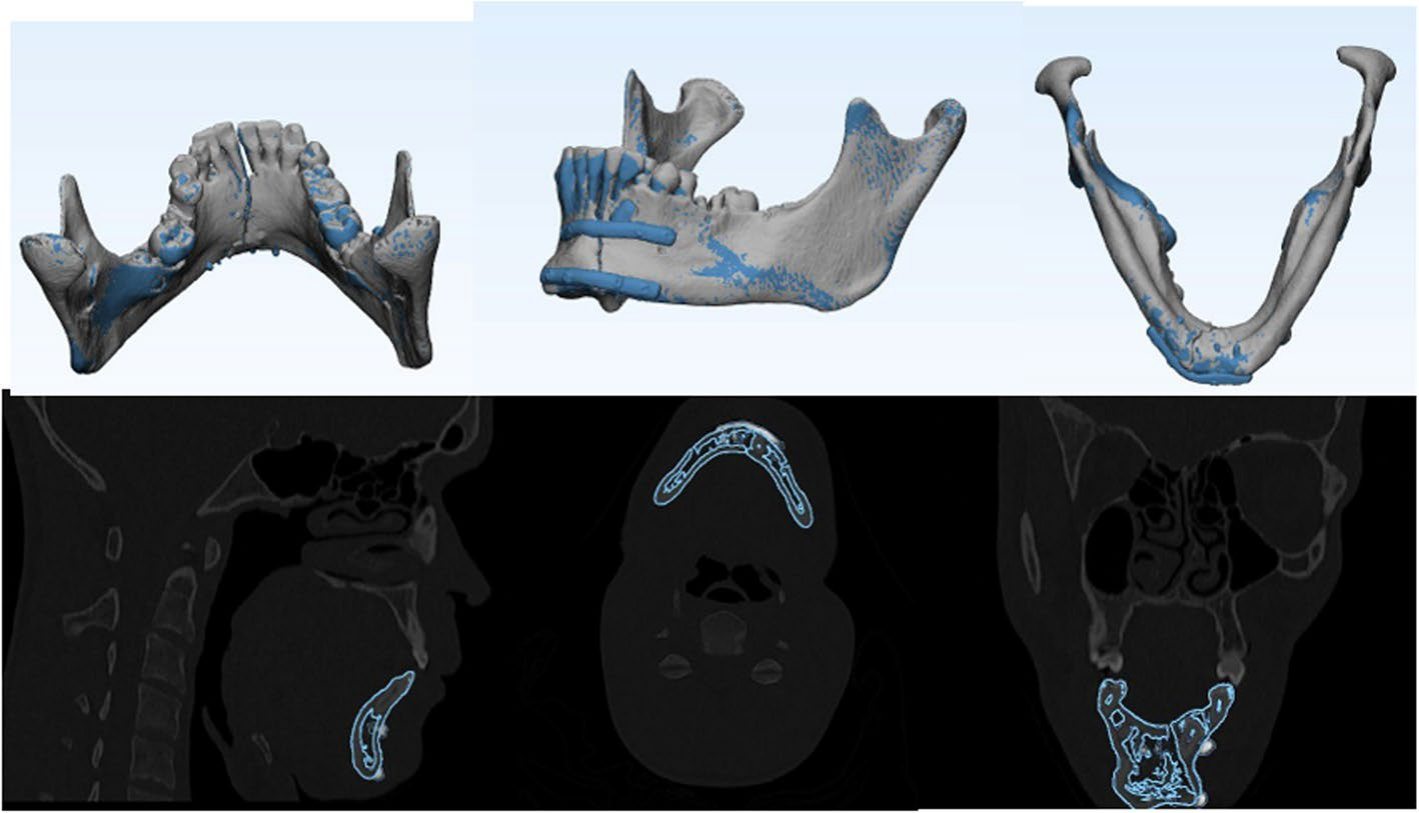
Case (2): Snapshot of multiplanar Mimics (Materialise NV) screen showing the post operative radiographic assessment (2D and 3D congruence) through super imposition of the actual postoperative CT model over the virtual surgical planning model in the study group

Within the first postoperative week, all enrolled participants underwent postoperative CT scan. The DICOM format were imported to Mimics 21.0 to create the actual postoperative mandibular model. Later this model was digitally superimposed onto the VSP model using an iterative closest point (ICP) algorithm and surface-based registration on Mimics 21.0, for 2D and 3D congruence (Figs. 23 and 24).

**Fig. 25:**
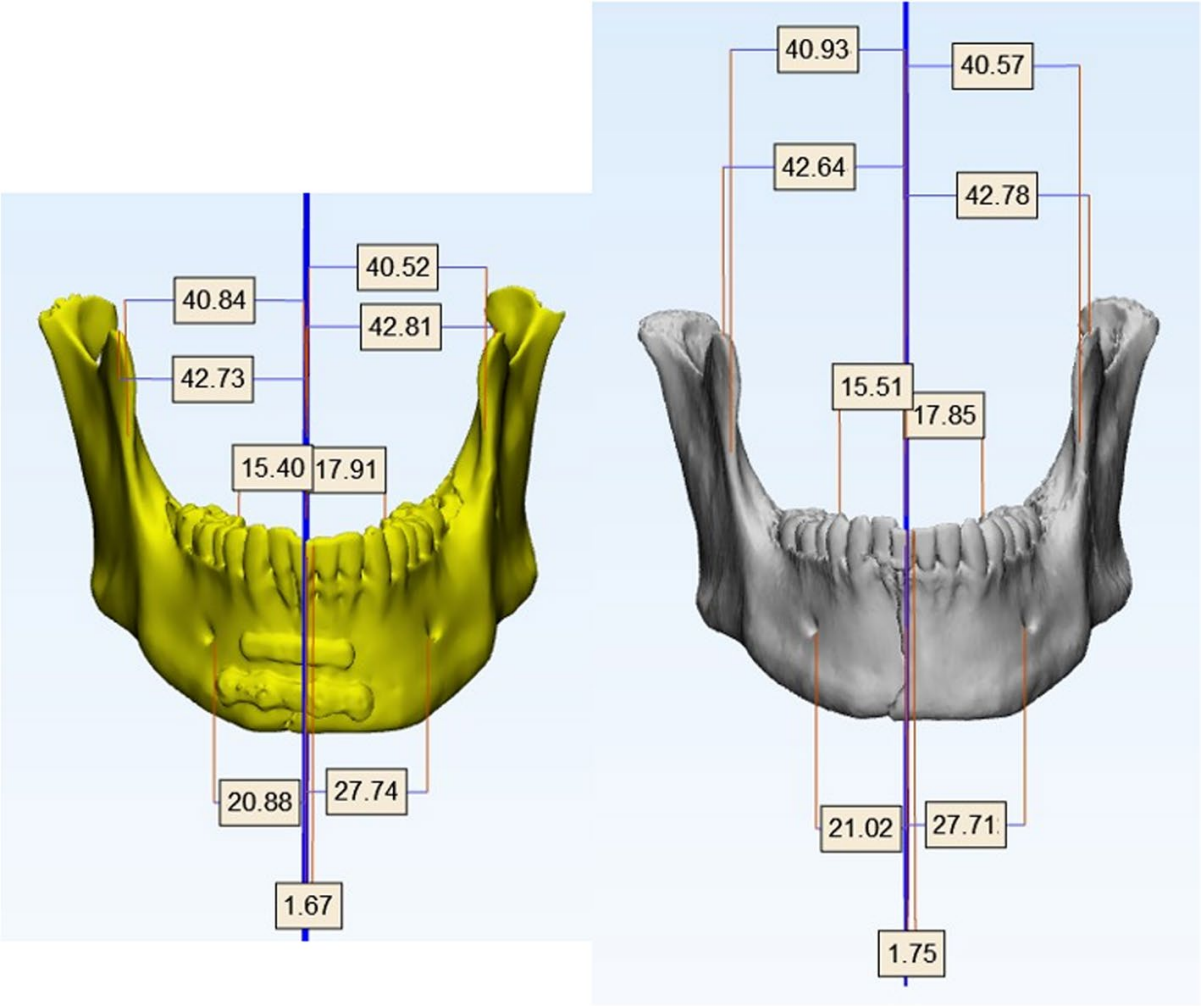
Case (1): Snapshot of multiplanar Mimics (Materialise NV) screen showing the absolute measure (in millimeters) of a perpendicular line from bony landmarks (coronoid tip bilaterally, lingula bilaterally and mental foramen bilaterally) and dental landmarks (centrals contact point and mesiolingual cusp tip of lower first molars bilaterally) to Mid-sagittal plane in the actual postoperative CT model (yellow model) and in the VSP reduced model (grey model)

**Fig. 26:**
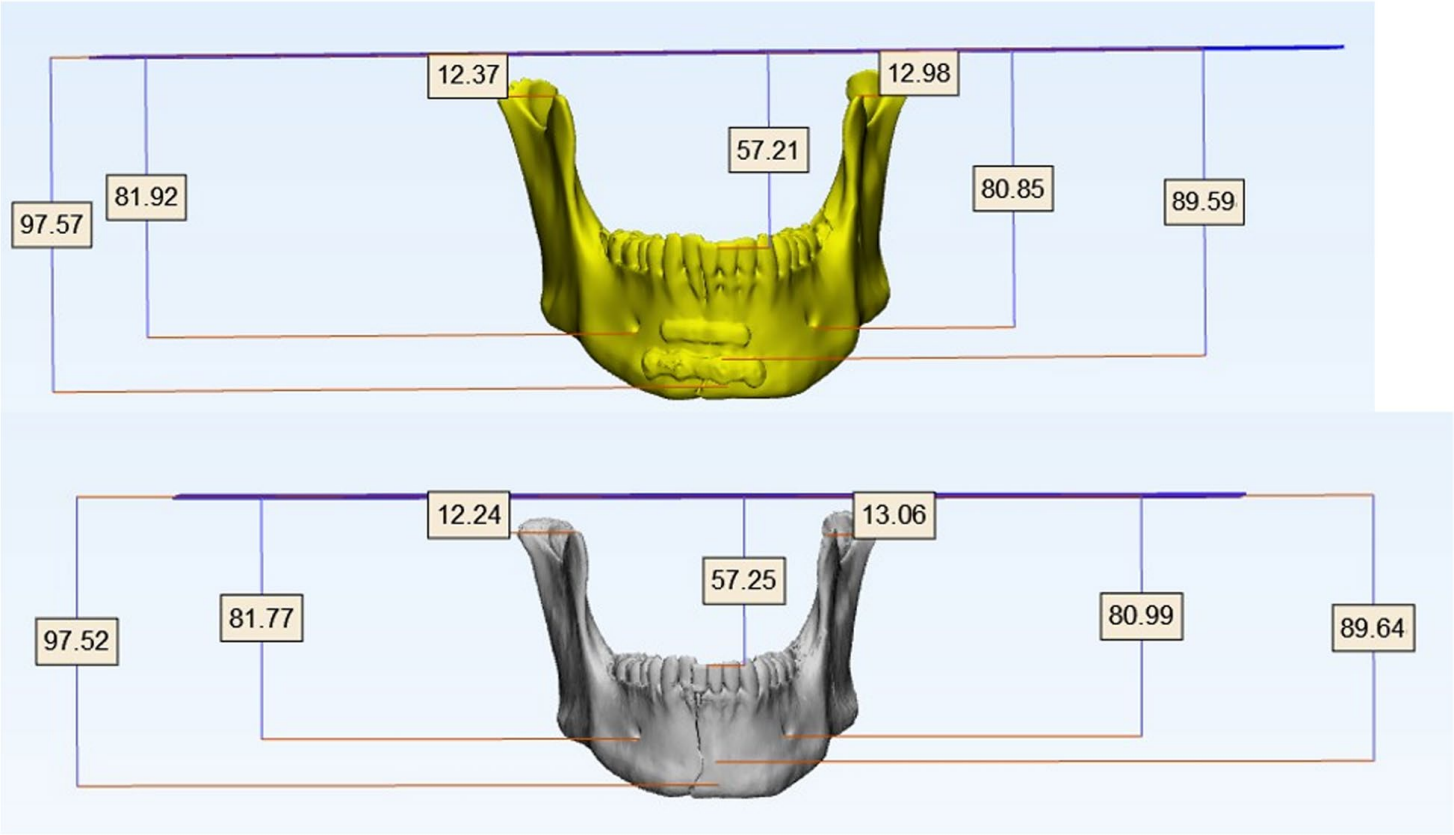
Case (1): Snapshot of multiplanar Mimics (Materialise NV) screen showing the absolute measure (in millimeters) of a perpendicular line from bony landmarks (coronoid tip bilaterally, mental foramen bilaterally, menton and pogonion) and dental landmark (centrals contact point) to Frankfort horizontal plane in the actual postoperative CT model (yellow model) and in the VSP reduced model (grey model)

**Fig. 27:**
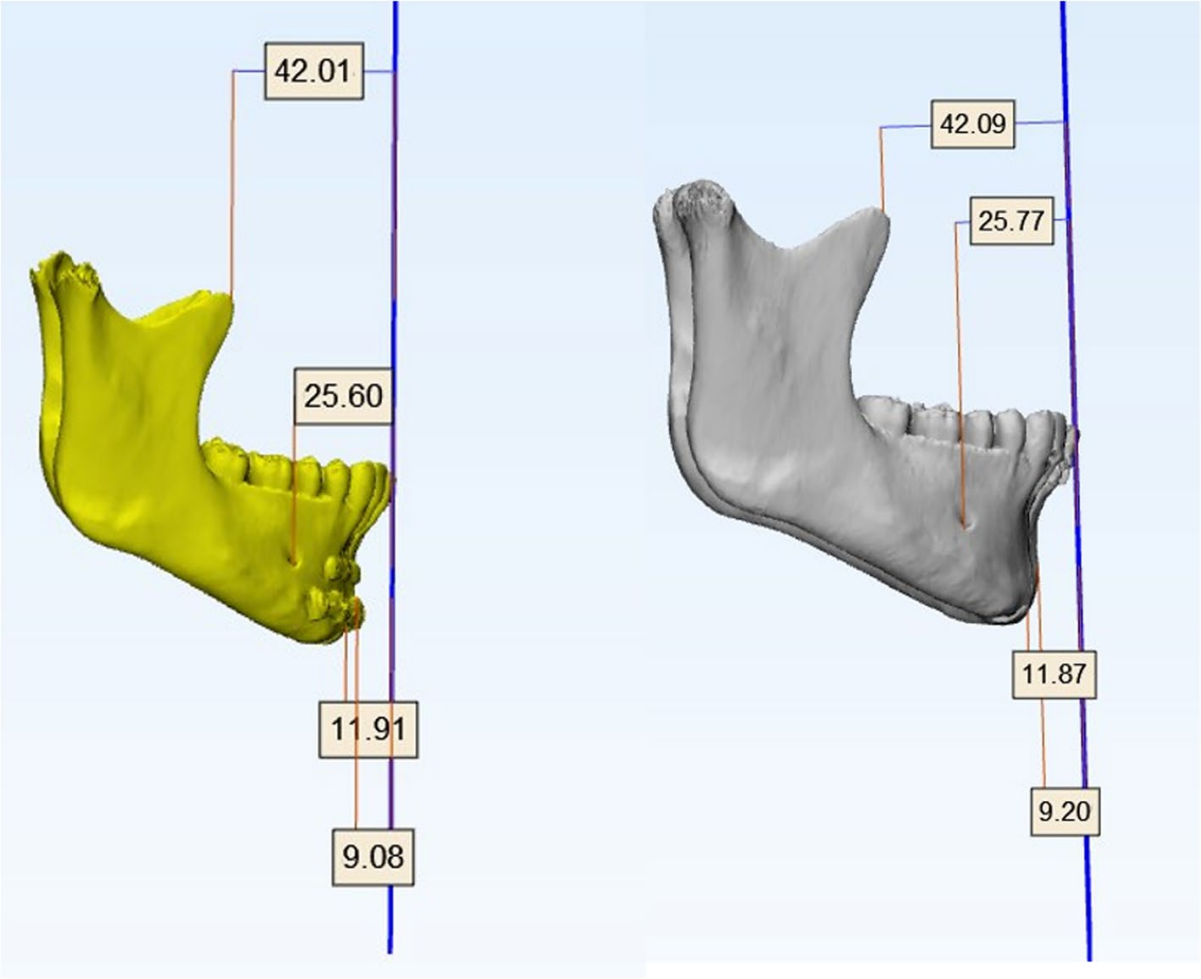
Case (1): Snapshot of multiplanar Mimics (Materialise NV) screen showing the absolute measure (in millimeters) of a perpendicular line from bony landmarks (coronoid tip bilaterally, mental foramen bilaterally, menton and pogonion) to coronal plane in the actual postoperative CT model (yellow model) and in the VSP reduced model (grey model)

The primary outcome was the precision of reduction, by measuring linear deviations of the actual postoperative model compared to the VSP model for both study and control groups. The deviations were calculated from absolute measure (in millimeters) of a perpendicular line from each predefined bony and dental landmark to each of the three predefined anatomical planes in the actual model derived from the postoperative CT subtracted from absolute measure of the same landmark and plane in the VSP reduced model (Figs. 25, 26 and 27).

Deviation = absolute distance between one landmark to one plane in the actual model derived from the postoperative CT − absolute distance between one landmark to one plane in the VSP reduced model.

All radiographic measures were evaluated by two assessors, and the final value was the average of the two readings. The resulting data were gathered and subsequently submitted for statistical analysis to obtain intragroup statistical analysis for study group, intragroup statistical analysis for control group, and intergroup statistical analysis between the two groups. Given the non-parametric distribution of the deviation data, the Wilcoxon signed-rank test (within-group) and the Mann–Whitney U test (between-group) were employed.

Bony landmarks were the coronoid tip bilaterally, mental foramen bilaterally, lingula bilaterally, menton, pogonion, and genial tubercles.

Dental reference points were the mesioincisal Contact Points of the lower central incisors and mesiolingual cusp tip of the lower first molar bilaterally. In cases with missing any lower first molars bilaterally, the mesiolingual cusp tip of the lower second molar—if intact bilaterally—followed by the mesiolingual cusp tip of the lower second premolar—if intact bilaterally—were served as a substitute dental landmark.

Both bony and dental landmarks were selected as surface registry point in Mimics 21.0 software in both the VSP model and the actual postoperative model. These reference points were selected based on their reproducibility and clinical relevance. All landmarks were identified independently by two trained assessors.

The spatial predefined planes were Frankfort horizontal plane (FHP): horizontal plane passing by the highest point of external auditory meatus bilaterally and lowest point of orbitale, while Mid-Sagittal plane (MSP): vertical plane passing by the nasion, sella turcica, and the midpoint of the anterior nasal spine, while Coronal plane (CP): vertical plane perpendicular to both FHP and MSP.

The FHP and MSP were constructed by creating datum plane, through 3 points on Mimics 21.0. separately, by selecting the corresponding 3 points in each plane. While the CP were constructed by creating datum plane, perpendicular to 2 planes (FHP and MSP) on Mimics 21.0.

#### Secondary outcome (occlusion)

The VSP reduced model was generated by the aid of the sectioned and realigned digital bite, which reflects the patient’s native occlusion. Therefore, the radiographic deviation of dental landmarks from the VSP model served as a quantitative, objective measure of occlusal accuracy.

Objectively, occlusion was evaluated by the precision of reduction, by measuring linear dental deviations of the actual postoperative model compared to the VSP model for both study and control groups. The deviations were calculated from absolute measure (in millimeters) of a perpendicular line from each predefined dental landmark to each of the three predefined anatomical planes in the actual model derived from the postoperative CT subtracted from absolute measure of the same landmark and plane in the VSP reduced model (Figs. 25, 26 and 27).

Deviation = absolute distance between one landmark to one plane in the actual model derived from the postoperative CT − absolute distance between one landmark to one plane in the VSP reduced model.

Clinically, occlusal was evaluated Visual inspection of maximum intercuspation, and by using articulating paper (8 μm, Bausch, Germany) at the following postoperative time intervals: 1, 2, and 4 weeks, to check for occlusal harmony and detect any premature contact.

### Surgical duration

Operative time was measured from the beginning of the incision to the completion of wound closure using a standardized operating room timer.

### Other outcomes

Wound healing, dehiscence, occlusion, and infection were assessed at the following postoperative time intervals: 1, 2, and 4 weeks.

### Sample size calculation

A power statical assessment was executed to attain sufficient statistical strength for a two-sided hypothesis test, with the null hypothesis positing no significant difference in reduction precision between the groups. The parameters were set with an alpha (α) of 0.05, a beta (β) of 0.2 (equating to 80% power), and an effective size (Cohen's *d* = 1.35), which was adopted from prior research [13]. The resulted computation determined a minimum required sample size of 20 participants (10 for each group). compensating the possible attrition throughout the follow up period, the sample size was enlarged by 25% resulting in a final enrolment target of 26 patients (13 per group). The power analysis was executed using G*Power software (version 3.1.9.7) [17]. All subsequent statistical evaluations were carried out using IBM® SPSS® Statistics (Version 26 for Windows).

The primary outcome used for calculation of the sample size was the precision of reduction, by measuring linear deviations of the actual postoperative model compared to the VSP model for both study and control groups. The deviations were calculated from absolute measure (in millimeters) of a perpendicular line from each predefined bony landmark to each of the three predefined anatomical planes in the actual model derived from the postoperative CT subtracted from absolute measure of the same landmark and plane in the VSP reduced model.

Deviation = absolute distance between one landmark to one plane in the actual model derived from the postoperative CT − absolute distance between the same landmark to the same plane in the VSP reduced model.

### Randomization

#### Sequence generation

All participants signed written informed consent before enrolment, randomly divided into two groups via a simple randomization procedure.

#### Allocation concealment

Sequentially numbered, closed, opaque envelopes (n = 13 per group) were prepared by an investigator not involved in the trial.

#### Implementation

Each participant drew a single envelope to reveal their assigned group designation: 1 for the study group or 2 for the control group. All 26 patients completed the trail and were included in the postoperative statistical analysis. No modifications to the trial protocol were made after study inception.

#### Blinding

The study was designed as a double-blinded controlled investigation where both the outcome assessor and the participant were masked.

#### Statistical methods

Categorical data (e.g., gender) are presented as frequencies and percentages and were analyzed using Fisher's exact test. Numerical data are presented as mean ± standard deviation (SD). Data normality was assessed using distribution plots (histograms and Q–Q plots) and the Shapiro–Wilk test. Given the non-parametric distribution of the deviation data, the Wilcoxon signed-rank test (within-group) and the Mann–Whitney U test (between-group) were employed. *P*-values were corrected for multiple comparisons using the false discovery rate (FDR) method, with statistical significance set at *p* < 0.05. All statistical analyses were conducted using R (version 4.4.2 for Windows; R Core Team, 2024). R: A language and environment for statistical computing. R Foundation for Statistical Computing, Vienna, Austria. URL: https://www.R-project.org/.

Interclass and intraclass correlations were calculated to assess agreement between 2 readings (either inter-observer and intra-observer) for continuous variables with the following classification; ICC values range from 0 to 1, classified as poor (< 0.5), moderate (0.5–0.75), good (0.75–0.9), or excellent (> 0.9).

## Results

This RCT was executed on 26 patients presenting with isolated, uncomminuted parasymphyseal mandibular fracture.

### Demographic data

The two groups were comparable with respect to age and sex distribution at baseline. An independent-samples *t*-test revealed no significant difference in mean age between the study group (27.31 ± 7.52 years) and the control group (29.38 ± 7.38 years); *t*(24) = 0.71, *p* = 0.484, Cohen's *d* = 0.28. Fisher's exact test showed no significant difference in sex distribution between groups (9 males [69.23%] and 4 females [30.77%] in the study group vs. 11 males [84.62%] and 2 females [15.38%] in the control group; *p* = 0.645) (Table 2).

**Table 2:** Intergroup comparisons of demographic data

Parameter	Study Group (*n* = 13)	Control Group (*n* = 13)	Test Statistic	*p*-value	Effect Size
Male	9 (69.23%)	11 (84.62%)	Fisher's exact	0.645	—
Female	4 (30.77%)	2 (15.38%)			
Age (years), Mean ± SD	27.31 ± 7.52	29.38 ± 7.38	t(24) = 0.71	0.484	d = 0.28

t(24) = t-statistic with 24 degrees of freedom; d = Cohen's d effect size. No significant differences were observed between groups

### Operative time

The intervention group demonstrated a significantly shorter mean surgical time (1.46 ± 0.18 h) in comparison to the conventional group (1.80 ± 0.34 h). A Mann–Whitney U test revealed a statistically significant difference between groups (U = 34.5, *p* = 0.004, *r* = 0.55) (Table 3).

**Table 3:** Intergroup comparisons and summary statistics for alignment time (hours)

Group	*M* ± *SD* (hours)	Test Statistic	*p*	Effect Size
Study Group (*n* = 13)	1.46 ± 0.18	*U* = 34.5	0.004	*r* = 0.55
Control Group (*n* = 13)	1.80 ± 0.34			

M = mean; SD = standard deviation; U = Mann–Whitney U test statistic; *r* = rank-biserial correlation effect size. The difference between groups was statistically significant (*p* < 0.05)

### Clinical outcomes

All cases in both groups showed uneventful wound healing excluding one patient in the conventional group (7.7%). This one case demonstrated wound dehiscence with plate exposure at the first postoperative week, which was managed with meticulous saline irrigation and antiseptic mouth wash. Complete healing was observed at the third postoperative week.

### Dental status

All 26 patients (*n* = 26) were dentate or partially edentulous with intact lower first molars bilaterally except for two cases in the interventional group and three cases in the conventional group. These five cases had intact lower second molars bilaterally which served as substitute dental landmarks. No patients required second premolar substitution. No patients were excluded due to asymmetric tooth loss or insufficient posterior dentition.

### Clinical occlusal outcomes

All patients demonstrated uneventful occlusal harmony with maximum intercuspation, excluding for two patients (15.4%) in the conventional group who showed immediate postoperative occlusal disharmony and premature contact, which necessitate heavy elastic application for 2–3 weeks. Finally, all patients attained stable maximum intercuspation by the first postoperative month.

### Intragroup radiographic comparisons for primary outcome (precision of reduction) and secondary outcome (occlusion)

Wilcoxon Signed-Rank Test was performed inside each group.

Control group (Table 4).

**Table 4:** Intragroup comparisons and summary statistics for the conrol group

Landmark	Plane	Mean ± SD (mm)		*p*-value
		Virtual reduction	Postoperative	
Bony	FHP	67.99 ± 32.16	68.72 ± 32.08	< 0.001*
	MSP	24.16 ± 18.73	24.79 ± 19.08	< 0.001*
	CP	39.77 ± 18.92	40.47 ± 19.09	< 0.001*
	Average	43.97 ± 6.19	44.67 ± 6.21	< 0.001*
Dental	FHP	66.42 ± 9.30	66.76 ± 9.22	< 0.001*
	MSP	12.38 ± 7.97	12.82 ± 8.13	< 0.001*
	CP	23.56 ± 13.09	24.19 ± 13.32	< 0.001*
	Average	34.12 ± 6.51	34.59 ± 6.65	< 0.001*

^*^ Significant (*p* < 0.05)

Study group (Table 5).

**Table 5:** Intragroup comparisons and summary statistics for the study group

Landmark	Plane	Mean ± SD (mm)		*p*-value
		Virtual reduction	Postoperative	
Bony	FHP	69.74 ± 32.03	69.77 ± 32.05	0.947(ns)
	MSP	24.72 ± 19.04	24.77 ± 19.05	0.965(ns)
	CP	41.75 ± 19.37	41.81 ± 19.38	0.914(ns)
	Average	45.41 ± 6.12	45.45 ± 6.13	0.970(ns)
Dental	FHP	68.59 ± 9.76	68.64 ± 9.76	0.844(ns)
	MSP	13.01 ± 8.48	13.05 ± 8.47	0.935(ns)
	CP	26.14 ± 14.49	26.18 ± 14.48	0.901(ns)
	Average	35.92 ± 7.24	35.96 ± 7.22	0.928(ns)

^*^ Significant (*p* < 0.05), ns not significant

**Fig. 28:**
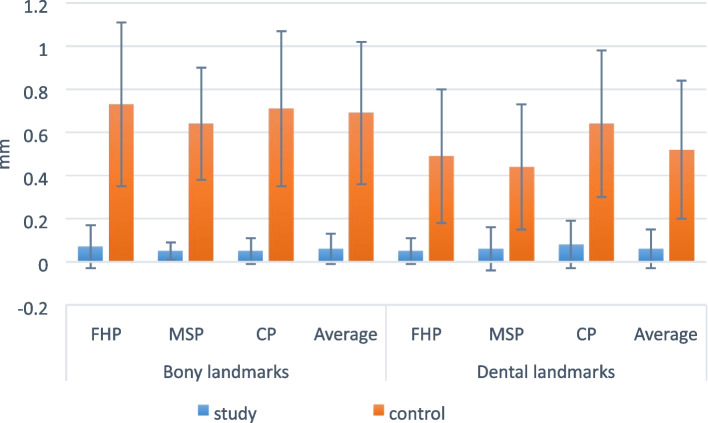
Bar chart showing mean and standard deviation for deviations measured in both groups

Intergroup radiographic comparisons for primary outcome (precision of reduction) and secondary outcome (occlusion) (Fig. 28) (Table 6).

**Table 6:** Intergroup comparisons and summary statistics for deviations

Landmark	Plane	Mean ± SD (mm)		*p*-value
		Study	Control	
Bony	FHP	0.04 ± 0.19	0.73 ± 0.58	< 0.001*
	MSP	0.04 ± 0.14	0.64 ± 0.70	< 0.001*
	CP	0.05 ± 0.15	0.71 ± 0.63	< 0.001*
	Average	0.044 ± 0.14	0.69 ± 0.50	< 0.001*
Dental	FHP	0.05 ± 0.14	0.34 ± 0.61	0.004*
	MSP	0.04 ± 0.12	0.44 ± 0.41	< 0.001*
	CP	0.04 ± 0.13	0.64 ± 0.50	< 0.001*
	Average	0.04 ± 0.13	0.47 ± 0.33	< 0.001*

^*^ Significant (*p* < 0.05)

A Mann–Whitney U test was performed between groups.

The mean bony landmark deviation relative to the FHP was 0.04 ± 0.19 mm in the intervention group, compared to 0.73 ± 0.58 mm in the conventional group. This difference was statistically significant (*p* < 0.001). Likewise, the mean dental landmark deviation relative to the FHP was 0.05 ± 0.14 mm in the intervention group versus 0.34 ± 0.61 mm in the conventional group (*p* = 0.004).

The mean bony landmark deviation relative to the MSP was 0.04 ± 0.14 mm in the intervention group and 0.64 ± 0.7 mm in the conventional group (*p* < 0.001). The mean dental landmark deviation relative to the MSP was 0.04 ± 0.12 mm in the intervention group compared to 0.44 ± 0.41 mm in the conventional group (*p* < 0.001).

The mean bony landmark deviation relative to the CP was 0.05 ± 0.15 mm in the intervention group versus 0.71 ± 0.63 mm in the conventional group (*p* < 0.001). The mean dental landmark deviation relative to the CP was 0.04 ± 0.13 mm in the intervention group and 0.64 ± 0.5 mm in the conventional group (*p* < 0.001).

The overall mean bony landmark deviation across all planes was 0.044 ± 0.14 mm in the intervention group and 0.69 ± 0.5 mm in the conventional group (*p* < 0.001). The overall mean dental landmark deviation was 0.04 ± 0.13 mm in the intervention group and 0.47 ± 0.33 mm in the conventional group (*p* < 0.001).

Inter-observer agreement illustrates excellent agreement between 2 observers for FHP, MSP and CP for bony readings with interclass correlation (ICC = 1., 0.997 &1.0, respectively). Also; there is excellent agreement between 2 observers for FHP, MSP and CP for dental readings with interclass correlation (ICC = 0.994, 1.0 &0.994, respectively) (Table 7). While, intra -observer agreement illustrates excellent agreement between 2 readings of same observer for FHP, MSP and CP for bony readings with interclass correlation (ICC = 1.0, 0.997 &0.992, respectively). Also; there is excellent agreement between 2 readings of same observer for FHP, MSP and CP for dental readings with interclass correlation (ICC = 0.997, 1.0 &1.0, respectively) (Table 8).

**Table 7:** Interobserver agreement of studied parameters

		ICC (95%CI)	*p* value
Bony	FHP	1.0(1.0–1.0)	*p* = 0.001*
	MSP	0.997(0.997–0.998)	*p* = 0.001*
	CP	1.0(1.0–1.0)	*p*= 0.001*
Dental	FHP	0.994(0.990–0.996)	*p* = 0.001*
	MSP	1.0(1.0–1.0)	*p* = 0.001*
	CP	0.994(0.990–0.999)	*p* = 0.001*

*ICC* Interclass correlation, *CI* Confidence interval

^*^statistically significant

**Table 8:** Intra -observer agreement of studied parameters

	post	ICC (95%CI)	*p* value
Bony	FHP	1.0(1.0–1.0)	*p* = 0.001*
	MSP	0.997(0.997–0.998)	*p* = 0.001*
	CP	0.992(0.990–0.999)	*p* = 0.001*
Dental	FHP	0.997(0.995–0.998)	*p* = 0.001*
	MSP	1.0(1.0–1.0)	*p* = 0.001*
	CP	1.0(1.0–1.0)	*p* = 0.001*

*ICC* Interclass correlation, *CI* Confidence interval

^*^statistically significant

## Discussion

Mandibular fractures constitute the most prevalent type of isolated facial fracture requiring surgical intervention, as evidenced by analyses of national and international surgical databases [18, 19]. Sufficient repair of mandibular fractures not only re-establishes an individual’s capability to speak, chew, breathe, and sleep but also restores patient’s native occlusion and facial aesthetics [5]. Although treatment modalities are well established, ongoing challenges associated with the classical workflow have been documented in previous studies, particularly in severely displaced fractures and in cases of partial or completely edentulous and atrophic mandible [4, 7]. This fracture’s pattern elevated the intraoperative difficulties to gain an accurate 3D anatomical reduction and optimal stabilization of the fracture segments [20, 21].

It is nearly impossible to contour a plate to achieve perfect intimate adaptation to the bone surface without any residual gaps. Intraoperative bending and adaptation of ready-made plates can develop significant, unpredictable, potentially detrimental internal stresses as well as strain hardening from cold working, and induced bending moments at the screw-bone interface [10, 22]. These stresses may lead to plate fatigue failure under cyclic loading of mastication, reduced plate lifespan as well as screw loosening. Consequently, screw loosening may lead to reduction in the fixation hardware, uneven load transfer and altered environment required for healing according to Wolff's Law, resulting in secondary bone healing with callus formation [23, 24].

In contrast, patient-specific custom-made plates milled from CP Ti-Grade 4 alloy represent the armamentarium for the stress-free fixation State, through passive adaptation without intraoperative contouring. There are no stress risers or cold working from bending. Although, Ti-Grade 4 has a lower ultimate tensile strength (550 MPa) compared to titanium Grade 23 (Ti-6Al-4V ELI) (900–1000 MPa), it offers excellent ductility, excellent fatigue resistance, enhanced fracture toughness, excellent biocompatibility and high corrosion resistance [10, 25]. In addition to, the custom-made plates ensure optimal load sharing. This creates the ideal mechanical environment for primary bone healing without callus formation and facilitates primary osseous healing across the fracture line [26].

The conventional plate osteosynthesis approach caries wide-scale limitations, and is associated with several complications. Narrow and restricted surgical access necessitates more tight flap retraction, leading to disruption of local vasculature, wound dehiscence with plate exposure, and infection [9]. This was consistent with the finding that one case in the conventional group showed wound dehiscence with plate exposure. In addition to, the conventional workflow relies on ancillary intraoperative techniques. These include supplementary intraoperative intermaxillary fixation with Erich arch bar or IMF screws, and intraoperative reduction by bone holding clamp. These techniques carry a notable risk of iatrogenic injury, including tooth avulsion, root damage, fracture of the buccal cortex and neurovascular bundle injury [7, 9]. The use of bone-holding clamp does not ensure complete reduction of the lingual cortex simultaneously with the buccal cortex along the fracture interface, leading to lingual flaring and suboptimal stabilization [27]. These ancillary techniques are less effective in partially edentulous cases lacking sufficient posterior occlusion pairs or completely edentulous cases [4, 9]. Furthermore, intraoperative contouring and adaptation of stock mini-plates is technique sensitive, time-consuming and highly reliant on surgeon’s experience.

On the contrary, the VSP protocol offers various advantageous over the conventional workflow. First, although wider initial surgical field seems more invasive, it facilitates precise placement of the screw-holes locator, more visibility and permits more gentle flap retraction, reducing soft tissue trauma [5]. Second, it eliminates the use of the supplementary intraoperative intermaxillary fixation with Erich arch bar or IMF screws, and reduction by bone holding clamp. It relies on the screw-holes locator and PSIs to translate the VSP the operative field. These custom-made plates maintaining the preplanned anatomical relationships, ensure a precise 3D anatomical reduction of the fracture segments including proper lingual cortex alignment and restoration of the patient’s native occlusion. Also, the precise fit and adaptation of these plates enhances overall biomechanical stability and fixation across the fracture interface minimizing the hazard of postoperative occlusal discrepancies [15]. Third, the VSP ensures safe and predetermined drilling trajectories with optimal screw predetermined lengths for each drill hole, minimizing the iatrogenic injury of adjacent anatomical structures. Fourth, while a one-piece guide is larger, it provides inherent rigidity and cross-arch stability with the aid of the vacuum-formed splint. It functions as an external fixator, maintaining the spatial relationship of the fragments as per the virtual plan during the drilling sequence. Accordingly, resulting in accurate 3D anatomical reduction using the predetermined drill holes for precise adaption and fixation of the custom-made plates. therefore, this methodology ensures the spatial coordinates for the custom-plate reduction and more predictable biomechanical stability of the fixation construct.

Clinically, all patients in the study group have experienced proper and almost perfect bone healing at the fracture sites without any complication. Therefore, this trial can be considered to be reflecting the clinical success and accuracy of a complete precise preplanned fixation strategy compared to the conventional techniques.

Vacuum-formed splint was used to stabilize the fractured segments by securing the dentition in their injury location during the preoperative CT acquisition. This splint was later utilized intraoperatively to restore and hold the fractured segments in a position identical to that established during the preoperative CT and virtual surgical planning, upon which the screw-holes locator was virtually constructed. The preservation of the fractured position ensured precise fitting and adaptation of the screw-holes locator to the fractured segments and facilitated the accurate translation of the VSP to the operating theatre. Vacuum-formed splint is not a reduction guide.

CT scans were acquired. the imaging dataset were stored in DICOM format and imported into specialized surgical planning software (Mimics, version 21.0; Materialise NV). Mimics (version 21.0; Materialise NV) software facilitates VSP by converting two-dimensional (2D) DICOM slices into a virtual 3D model through segmentation by detecting and identifying the anatomic architecture of the fracture mandible derived from the initial CT scan. Digital image segmentation with an appropriate threshold was performed to isolate the bony architecture, creating a precise 3D visualization of the fracture segments. The generated 3D fractured mandibular model undergone VSP with the aid of the composite skull-dental models and the predetermined digital bite registration to create a virtually reduced 3D mandibular model with proper anatomical reduction and occlusal harmony.

Composite skull-dental model was created by superimposing the laser-scanned dental models over the virtual fractured segments of the skull model. This Composite skull-dental model will be translated and superimposed over the predetermined digital bite registration to generate a reduced 3D virtual mandibular model. Virtual surgical planning reduction is the translation and superimposition of Composite skull-dental model over the predetermined digital bite registration to generate a reduced 3D virtual mandibular model.

Reverse engineering is a digital step, where the exact screw holes imprint coordinates from the designed plates over the reduced model were then transported to the fracture segments in the preoperative position to fabricate the screw-holes locator. This digital step ensures a precise fitting of the screw-holes locator to the fractured segments and accurate coordination of the drill holes for subsequent custom-made plate reduction and fixation in accordance with the VSP.

There have been efforts to integrate 3D printing technology combined with VSP. Thereby, Numerous researches have employed 3D software for VSP in the craniomaxillofacial field (CMF) field, which involves the fabrication of customized surgical guides [11–15, 28].

Prior protocol included 3D printing of a corrected anatomical model for plate contouring and fabrication of screw-holes locating guide through analogue reverse engineering technology. This guide delineated the accurate screw holes imprint coordinates on the fractured segments for subsequent pre-bend plate fixation [16]. That previous protocol was non fully digital and demonstrated various technical limitations, involving time consuming, complex, and sequential processes associated with physical model fabrication. These included 3D printing of the reduced mandibular model with its inherent risk of resin shrinkage or dimensional inaccuracies, manual bending and fixation of ready-made plates on the corrected model, laser scanning of the composite pre-bent plates–reduced mandibular model, subsequent superimposition for further virtual reverse engineering steps, and the manual allocation of screw-hole imprints within the software.

In contrast, our present methodology was designed to circumvent the inherent inaccuracies and inefficiencies of conventional plate adaptation and contouring even pre-bended on reduced printed model. Thus, it adopted the use of custom-made plates. In addition to, it offers a digital reverse engineering protocol that eliminates these physical sequential steps and their related inaccuracies, thereby preventing the propagation of domino toppling errors and enhancing overall surgical accuracy. Consequently, the radiographic statistical analysis revealed superiority at anatomical reduction and occlusion compared with the findings of Gaber et al. (2025) [16]. However, the workflow remains hybrid. The overall precision of this hybrid technique may be influenced by variables inherent to the analog components, including impression fidelity, stone cast accuracy, and splint fit.

In the current study, the use of PSIs were associated with an improved surgical workflow. This approach allowed for precise anatomical application of the plates prior to surgery, and eliminating the need for intraoperative contouring. These findings are consistent with those of Cho et al. (2023) [15], who reported that the proposed methodology achieved the desired patient-specific surgical outcome with minimal error. They conclusively demonstrated that custom-made plates designed on virtual 3D-reduced anatomical models provide superior adaptation with zero gaps to the buccal cortex across the fracture interface, compared to intraoperative manual contouring of ready-made plates. Such precise fit enhances the stability of the fixation construct and promotes more predictable reduction outcomes.

Concerning all predefined bony and dental reference points and planes, the postoperative assessments showed distinctive superiority in the study group utilizing patient-specific surgical screw-holes locator and custom-made plates osteosynthesis, compared to conventional workflow for mandibular fractures repair. These deviations are negligible when compared to the clinically acceptable deviation range of 2 mm.

Even though a 2 mm postoperative deviation is frequently cited as an acceptable threshold in maxillofacial trauma. Such deviation has a significant impact on clinical practice, posing substantial challenges for patient recovery and compliance. The resulting occlusal disharmony may often require more than two weeks of postoperative rehabilitation through complementary IMF or intermaxillary elastics to re-establish the patient’s native occlusion [29, 30]. This cascade effect shows that even minor rotational discrepancies along the fracture interface can be amplified into significant angular deviations in dental occlusion, consequently leading to occlusal discrepancies that compromise the overall stability of the fixation hardware, as well as functional and aesthetic outcomes [31].

The computer-guided protocol proposed in the study by El-Gengehi et al. [12] has limitations, as it focused solely on fracture segment immobilization using a custom surgical guide fabricated on a virtually reduced 3D mandibular model, followed by adaptation and fixation of ready-made titanium plates identical to those used in conventional plate osteosynthesis. The reported postoperative deviation range relative to MSP was 0.39 mm (SD), which is lower than the MSP deviation observed in the conventional group of the present study and substantially lower in accuracy compared to our study group.

A similar limitation was noted by Abdelaal et al. [13], who reported even higher mean MSP deviation values (2.4 mm). Their study adopted comparable computer-guided reduction methods but lacked a computer-assisted approach for the placement and adaptation of fixation hardware. Based on these comparative findings, the accuracy of computer-guided methodology is effectively enhanced when the protocol integrates a complete digital workflow, including the design and fabrication of patient-specific custom-made plates rather than relying solely on reduction guides while using standard stock ready-made plates, as in the conventional workflow.

Furthermore, their results relied solely on bony landmarks, and their postoperative linear measurements were compared to the VSP based only on the MSP. Therefore, these protocols showed only a two-dimensional assessment of anatomical reduction within only one plane, lacking spatial 3D evaluation of reduction accuracy and neglecting dental landmarks, and subsequent occlusal relationship assessment [12, 13].

In stark contrast, the current study offers methodological advantages superior to previous protocols. It not only employs a hybrid (digital-analogue) workflow but also utilized both predetermined dental and bony landmarks, and their postoperative linear deviations measured across the three anatomical planes. This enables comparison of actual surgical outcomes with the VSP for subsequent statistical analysis. Therefore, this protocol ensures a clinically relevant spatial evaluation of anatomical reduction and occlusal reestablishment.

The statical assessment in this study showed that the postoperative mean deviations of the predefined dental reference points across the three anatomical planes were closely matched the corresponding measurements obtained from the computer-guided reduced model. No statistically significant difference was observed between the two values, proving a high degree of fidelity between the virtual surgical plan and the executed surgical outcome. This quantitative validation reinforces the accuracy and reliability of the integrated computer-guided protocol utilizing a screw-hole locator and patient-specific custom-made plates for reestablishment of the patient’s native occlusion.

In contrast, the postoperative mean deviation of the predefined dental reference points across the three anatomical planes in the conventional group fluctuated from the measurements acquired from the VSP model. A statistically significant difference was observed, showing a measurable loss of occlusal precision inherent to the conventional technique. Clinically, reflected in two cases from the conventional group, who demonstrated occlusal disharmony and required heavy elastic application.

The VSP approach resulted in a statistically significant reduction in operative time. This benefit is likely due to its ability to allow rapid and precise three-dimensional realignment of fracture segments, along with the use of screw-holes locator and patient-specific plates. Together, these elements minimize time-consuming intraoperative procedures such as manual reduction and plate contouring. These results align with those reported by Gaber et al. (2025), who found that patient-specific screw-hole locating guide significantly reduce operative time in the management of Class III mandibular fractures, highlighting the role of digital planning and guided execution in improving surgical workflow [16].

Although plate designs varied to accommodate patient-specific anatomy (contour, length, and screw hole spacing), these adaptations were performed within a uniform digital workflow by a single author. While such design variations may influence biomechanical behavior, they did not significantly affect the primary clinical outcomes in this study. Future studies with larger sample sizes and biomechanical testing are needed to isolate the effect of individual design parameters. Consequently, the observed improvements in reduction precision, operative time, and occlusal outcomes cannot be attributed exclusively to any single element (e.g., the screw-hole locator alone or the custom-made plates alone). Rather, these benefits likely arise from the synergistic effect of the complete hybrid (digital-analogue) workflow, in which each component supports the others to achieve a precise, pre-planned, and efficiently executed reduction. This study was designed as a pragmatic comparison of two established treatment protocols, not as a component-by-component deconstruction of the digital workflow.

In essence, ready-made plates continue to represent a reliable and efficient treatment option. While custom-made plates provide a more precise solution for complex cases. As technology advances and becomes more widely available, the use of patient-specific implants is likely to increase. However, traditional miniplates will continue to play a crucial and irreplaceable role in the trauma surgeon’s toolkit for the foreseeable future [32, 33].

In summary, screw-holes locator combined with patient-specific plate osteosynthesis enable more precise management of parasymphyseal mandibular fracture, ensuring precise segment reduction and occlusion compared to the conventional workflow, as indicated by the resulted clinical and radiographic outcomes.

### Study limitations

The study was limited by the technique-sensitive, higher cost, availability, and lack of designing, milling infrastructure, and expert designers. Despite, the complete digital and analogue workflow from dental impressions and CT import to design and STL export of guides and plates required only 24 h. The actual production and delivery of sterilized devices required additional 1–2 working days.

The sample size -despite being statistically powered- is relatively small, which demonstrates a pilot-scale investigation. Therefore, a larger-scale trials are required to assess the generalizability of these findings.

The Current study’s inclusion criteria included only uncomminuted fractures, with dentate cases or partial edentulous cases with intact lower central and sufficient posterior occluding pairs. Regarding computer-guided mandibular fracture repair, future research recommendations should expand patient populations including communicated fractures, cases with atrophic mandible, and cases with segmental bone loss.

The study compares two complete treatment protocols rather than isolating a single variable. The superior precision in both radiographic and clinical results in the study group, cannot be attributed to a single element of the intervention but rather to the entire computer-guided virtual surgical planning protocol as a whole. Future researches using isolated components analysis, are required to isolate and attribute the relative contribution of each element in the computer-guided workflow.

Despite our study exceeds several prior VSP trials by including objective occlusal assessment (articulating paper), subjective occlusal evaluation, and 1 month follow-up data. We acknowledge that functional outcomes (bite force, TMJ function), long-term stability, complication rates, need for reintervention, and long-term follow-up to evaluate stress shielding phenomenon and screws loosening, represent the next frontier for VSP research.

## Conclusions

The proposed entire Computer-guided technique, incorporating patient-specific screw-holes locator and custom-made plates significantly enhances the precision and efficiency of parasymphyseal mandibular fracture reduction and fixation, with a significant reduction in operative time. This approach also highlights how virtual surgical planning can minimize reliance on surgeon experience and skill, subsequently ensuring more standardized and reproducible results. These benefits should be attributed to the synergistic effect of the entire hybrid workflow rather than to any single component.

## Data Availability

All data are available whenever requested from the corresponding author of the manuscript. Email: [ahmed.tageldeen@dentistry.cu.edu.eg](mailto:ahmed.tageldeen@dentistry.cu.edu.eg).

## Abbreviation

&: And
RCT: Randomized Controlled Trial
SD: Standard Deviation
OPG: Orthopantomogram (panoramic X-ray)
VSP: Virtual surgical planning
CT: Computed Tomography
PSI: Patient Specific Implants
PRS: Protocol Registration and Results System
3D: Three-dimensional
ORIF: Open Reduction and Internal fixation
STL: Stereolithographic
Mm: Millimeter
CMF: Craniomaxillofacial
Ti: Titanium
CP Ti: Commercially Pure Titanium
Ti-6Al-4V ELI: (Titanium, 6%Aluminum, 4%vandium, Extra Low Interstitial
MPa: Mega (one million) Pascal (pressure or stress unit defined as one Newton of force per square meter)
CNC: Computer Numerical Control System
FHP: Frankfort horizontal plane
MSP: Mid Sagittal Plane
CP: Coronal Plane
IMF: Inter-maxillary fixation
CAD/CAM: Computer aided design/computer aided manufacturing
DICOM: Digital Imaging and Communications in Medicine
CONSORT: Consolidated Standard of Reporting Trials
ICP: Iterative closest point
